# Collection and detection of SARS-CoV-2 in exhaled breath using face mask

**DOI:** 10.1371/journal.pone.0270765

**Published:** 2022-08-18

**Authors:** Hwang-soo Kim, Hansol Lee, Junsoo Park, Naseem Abbas, Seonghui Kang, Hakjun Hyun, Hye Seong, Jin Gu Yoon, Ji Yun Noh, Woo Joo Kim, Sehyun Shin

**Affiliations:** 1 Department of Micro-nano System Engineering, Korea University, Seoul, Republic of Korea; 2 Asia Pacific Influenza Institute, Korea University College of Medicine, Seoul, Republic of Korea; 3 Department of Mechanical Engineering, Sejong University, Seoul, Republic of Korea; 4 Division of Infectious Diseases, Department of Internal Medicine, Konyang University Hospital, Daejeon, Republic of Korea; 5 Division of Infectious Diseases, Department of Internal Medicine, Korea University College of Medicine, Seoul, Republic of Korea; 6 School of Mechanical Engineering, Korea University, Seoul, Republic of Korea; University of South Carolina, UNITED STATES

## Abstract

Face masks are used to protect the wearer from harmful external air and to prevent transmission of viruses from air exhaled by potentially infected wearers to the surrounding people. In this study, we examined the potential utility of masks for collecting viruses contained in exhaled breath and detected the collected viruses via various molecular tests. Using KF94 masks, the inner electrostatic filter was selected for virus collection, and an RNA extraction protocol was developed for the face mask. Virus detection in worn mask samples was performed using PCR and rolling circle amplification (RCA) tests and four different target genes (N, E, RdRp, and ORF1ab genes). The present study confirmed that the mask sample tests showed positive SARS-CoV-2 results, similar to the PCR tests using nasopharyngeal swab samples. In addition, the quantity of nucleic acid collected in the masks linearly increased with wearing time. These results suggest that samples for SARS-CoV-2 tests can be collected in a noninvasive, quick, and easy method by simply submitting worn masks from subjects, which can significantly reduce the hassle of waiting at airports or public places and concerns about cross-infection. In addition, it is expected that miniaturization technology will integrate PCR assays on face masks in the near future, and mask-based self-diagnosis would play a significant role in resolving the pandemic situation.

## Introduction

Since the outbreak of the coronavirus disease 2019 (COVID-19), infection with the severe acute respiratory syndrome coronavirus 2 (SARS-CoV-2) has spread worldwide, with more than 522 million infected cases, with 6.27 million deaths by May 2022. Transmission models indicate that rapid and accurate identification of SARS-CoV-2 and early diagnosis can greatly help control the pandemic [1]. This tremendous spread rate is mainly due to virus transmission via respiratory aerosols and droplets between asymptomatic infectors and infectees [2, 3]. It has been shown that small respiratory aerosols spread to distances more than 1 m from infectors when they exhale, and indoor ventilation may increase the traveling distance [4]. Thus, the virus-blocking efficacy of masks has not only been scientifically studied [5] but has also become a global issue owing to the mandatory wearing of masks in society.

In general, face masks have been used to protect the wearer from harmful external air and to prevent transmission of viruses from air exhaled by potentially infected wearers to the surrounding people [5]. Certified masks, such as the N95 grade, are strongly recommended for either protecting the wearer from external viral aerosols or blocking the release of viruses contained in the exhalation of the wearer. The N95 masks consist of four-layered membranes that utilize mechanical and electrostatic filtering in the mid-layered membranes [6]. One of the membranes uses permanently charged electret fibers as the filtering medium, and in vitro experiments have revealed that they collect > 95% aerosolized virus [7]. Owing to their performance characteristics N95 masks successfully block aerosols containing SARS-CoV-2 [5]. In other words, the viruses shed through exhaled breath are effectively collected in the facial masks.

Since the outbreak of COVID-19, wearing face masks has become part of daily life in all countries. For better safeguard against coronavirus, official agencies such as FDA and the Centers for Disease Control and Prevention (CDC) have strongly recommended to use the certified grade level mask such as KN95, N95 and KF94 masks (so called respirators) rather than fabric mask. The National Institute of Occupational Safety and Health (NIOSH) tests and approves air respirators performance of particulate filtering. The minimal level of filtration approved by NIOSH is 95 percent. All of KN95, N95 and KF94 masks filter out particles as small as 0.3 μm. For instance, N95 masks filter 95 percent of airborne particles, whereas KF94 masks meet the South Korea’s safety standards and filter 94 percent of particles. Many of KF94s are also FDA approved. Thus, there is no major difference between N95 and KF94.

It has been reported that breath sampling using a face mask can be used to effectively collect pathogens in a minimally invasive manner [8]. Studies have shown that early-stage infected patients release large numbers of SARS-CoV-2 particles through breathing, coughing, talking, or sneezing [1]. Virus sampling using nasopharyngeal swabs is currently the standard protocol, requiring a rather tedious process and long queues for public sampling. Unlike nasopharyngeal swabs, breath sampling does not require trained medical personnel or privacy, does not generate potentially infectious waste, and can be performed in essentially any time frame. Submitting a mask worn over a period in a sanitary envelope, as a new virus capture protocol, could overcome the limitations of the current sampling method.

Therefore, there is an urgent need to examine the potential utility of exhaled breath sampling with a mask for virus detection. Recent studies [9–11] reported that SARS-CoV-2 viral RNA was detected and quantified using RT-PCR in the mask. In addition, facial masks were further integrated with sensors for the direct detection of viruses collected from exhaled breath [12, 13]. Recently, we reported rapid diagnosis of SARS-CoV-2 using DNA hydrogel formation on microfluidic pores, which yielded a limit of determination (LOD) (0.7 aM) equivalent to that of conventional PCR [14]. Despite the advances in related research, researchers have encountered technical challenges in the following aspects: 1) collection of respiratory viruses from masks without contamination; 2) extraction of viral RNA from the masks; and 3) detection of targeted viruses. Although each of them may raise many issues, the present study focused on the utility and potential of mask-based diagnosis of infectious viruses. Thus, we designed a systematic study consisting of 1) virus collection with a newly designed mask, 2) quantification of extracted RNA, and 3) virus sensing with various genes (N, E, RdRp, ORF1ab, and internal control) using PCR and RCA. With careful examination of each topic, we investigated the potential use of facial masks as a new virus sampling method.

## Methods and materials

All procedures performed in this study involving human participants were in accordance with the ethical standards of the institutional and/or national research committees and with the standards of the 1964 Helsinki Declaration and its later amendments or comparable ethical standards. The study protocol was approved by the Institutional Review Board of the Korea University Guro Hospital (approval No.:2021GR0092) and the Konyang University Hospital (approval No.: KYUH 2020-12-018-003). The materials used in this study are described in S1 File. Throughout this study, all experiments were performed in five replicates (n = 5) for each condition.

### Sample collection and experimental setup

We collected facial masks from SARS-CoV-2 infected patients who were identified though official PCR tests at Korea University Guro Hospital and the COVID-19 living treatment center operated by Konyang University Hospital. For comparison, masks from the negative controls were also collected. With their written consent, these masks were carefully collected in a sterilized vinyl bag and immediately preserved in a cold freezer at -80°C. The sample masks were collected from the masks worn by patients within 1 to 7 days from the date of confirmed covid-19. Samples that did not meet the exclusion criteria were excluded from this study. The exclusion criteria for sample collection and storage are 1) if it is not a mask worn by a confirmed COVID-19 patient; 2) when the mask wearing time is insufficient (t > 4 hr); and 3) if the storage temperature (< 80°C) is not satisfied after sample collection.

**Fig 1:**
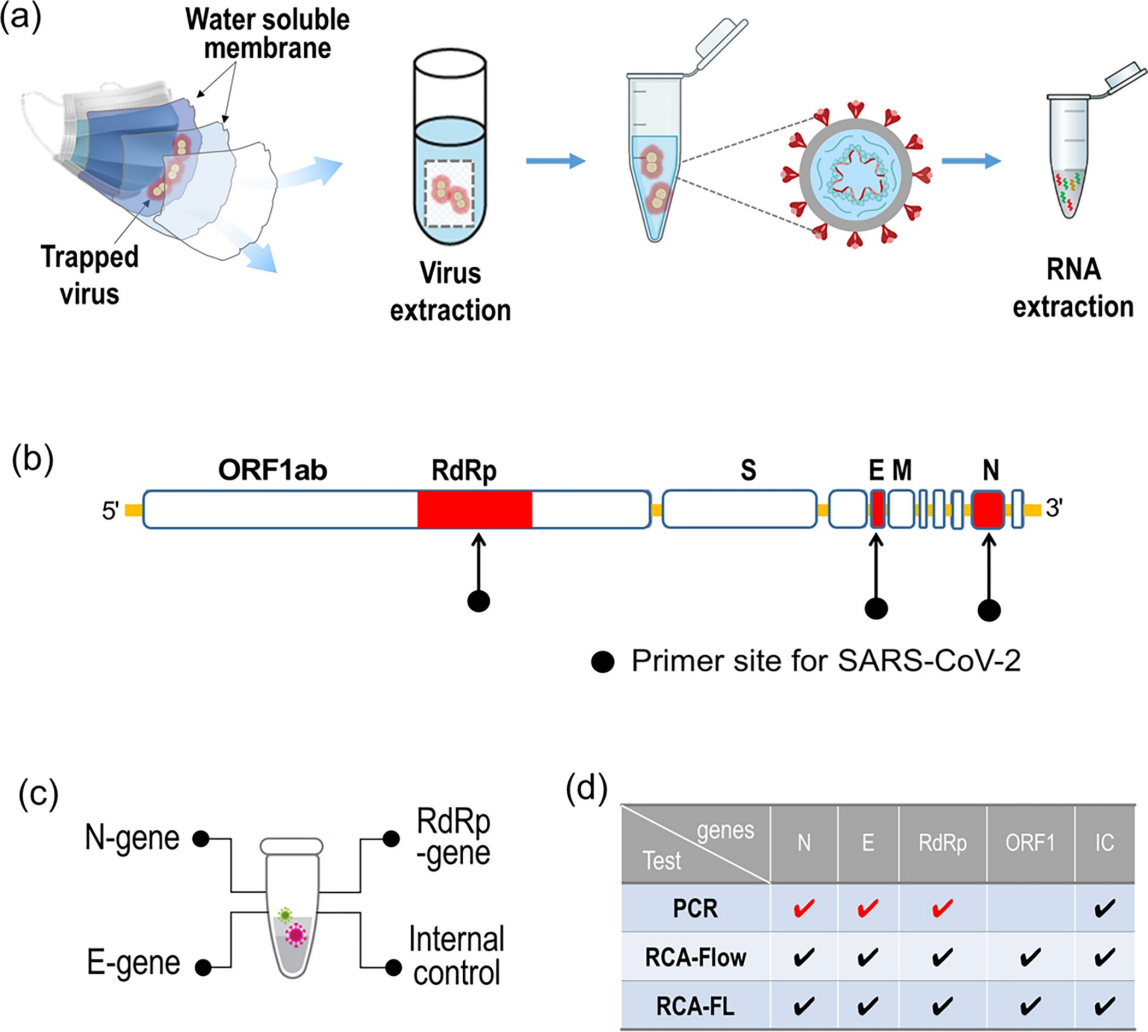
**(**A) Process of viral RNA extraction from a mask in exhalation including virus collection with a mask from exhaled breath, extraction of virus from mask membranes, isolation of viruses; and extraction of viral RNA; **(**B) Primer sites to identify SARS-CoV-2; **(**C) SARS-CoV-2 target genes extracted from mask; **(**D) List of target genes for three different assays including PCR, RCA-Flow and RCA-FL.

In Fig 1(A), the process of viral RNA extraction from the mask is briefly depicted. To identify SARS-CoV-2, we adopted four target genes, N-, E-, RdRp-, and ORF1ab-genes, as shown in Fig 1(B) and 1(C). These target genes have been used in many commercial assays. For instance, we adopted a PCR test by purchasing commercial PCR reagents from Seegen, which used the N-, E-, and RdRp genes as target genes. As shown in Fig 1(D), we adopted two additional tests based on RCA: RCA-Flow and RCA-FL. The former test is a rapid test using DNA hydrogel formation on microfluidic pores [14, 15], whereas the latter is a general RCA test with detection by fluorescence using a PCR device.

### Extraction of viral RNA prep from masks

All facial masks were KF94 certified and US Food and Drug Administration (FDA)-approved. Since the number 94 refers to its filtration efficiency (basically, how good the mask is at filtering out undesired particles), KF94 is comparable to N95, at least in blocking SARS-CoV-2 particles [16]. As depicted in Fig 1(A), a KF94 mask consists of four layers: an outer layer (nonwoven), a middle layer (nonwoven structural layer), a filter layer (electrostatic melt-blown layer), and an inner layer (nonwoven skin-friendly layer). Among these layers, we carefully chose the filter layer to collect the viruses while avoiding any contamination from the skin of the wearer and the external environment. The central area of the filter layer was cut to a square of size 50 mm × 50 mm and stored in a presterilized vinyl bag (Ziploc Double Zipper Freezer Bags, S C Johnson & Son Inc., 1525 Howe Street, Racine, Wisconsin, USA).

### Extraction of viral RNA prep from masks

The process of extracting RNA from the mask is described below. First, a cut mask membrane was placed in 5 mL of Trizol, incubated for 10 min at room temperature (24°C), and vortexed; then the mask was drained of all the solution using a syringe. Next, 200 μL of chloroform was dispensed into 1.5 mL tubes, and 1 mL of the mixture extracted from the mask was added. The mixture was shaken for 15 s and incubated at room temperature for 5 min. After centrifugation at 12,000 × *g* and 4°C for 10 min, only the transparent supernatant was separated. After mixing 500 μL of isopropanol with the supernatant, the mixture was incubated for 10 min at room temperature after stirring. The sample was centrifuged again at 12,000 × *g* and 4°C for 10 min, and then the supernatant was removed. After adding 1 mL of 75% ethanol to the RNA from which the supernatant was removed, the mixture was centrifuged at 7,500 × *g* and 4°C for 5 min, and then the supernatant (ethanol) was removed. Next, the mixture was dried (remove ethanol completely) for 5–10 min with the lid open and washed twice depending on the sample. Finally, after dispensing 15 μL of RNase-free water (DW) and pipetting, the mixture was incubated at 65°C for 10 min in a heating block and then incubated on ice for 2 min to extract RNA.

### PCR test with mask samples

In the present study, we purchased commercial test kits for SARS-CoV-2 (Allplex™ SARS-CoV-2 Assay, Seegene, Seoul, Korea), which received emergency use authorization from the FDA for use as a (COVID-19) diagnostic kit. This product is a real-time gene amplification (RT-PCR)-type COVID-19 diagnostic kit that detects all three target genes (E, RdRp, and N) to confirm SARS-CoV-2 infection. In Korea, it has also been approved for emergency use by the Ministry of Food and Drug Safety.

Allele-specific amplification was performed using a Real-Time PCR system (CFX96 Touch™ Real-Time PCR, Bio-Rad) as follows: First, the reaction master mix is prepared. The master mix contained 5 μL SARS2 MOM, 5 μL EM85, 5 μL RNase-free water, and the total volume of the master mix was 15 μL. The total amount of each reagent needed was calculated based on the number of reactions, including samples and controls. The master mix was vortexed and briefly centrifuged. Fifteen microliters of the reaction master mix was added to 0.1 mL (MicroAmp™ Fast 8-Tube Strip, 0.1 mL, Applied Biosystems™) 8-tube strip. Five microliters of the nucleic acids of each sample was added into the tube containing the reaction master mix. The 8-strip cap (MicroAmp™ Optical 8-Tube Cap Strip, Applied Biosystems™) was closed, and the PCR tubes were briefly centrifuged. The liquid containing all PCR components was at the bottom of each PCR tube. The thermal profile was set as follows: 1 cycle for 20 min at 50°C, 1 cycle for 15 min at 95°C, and then 45 cycles at 95°C for 10 s, 60°C for 15 s, and finally at 72°C for 10 s. The data were analyzed using Bio-Rad CFX Maestro software and the cycle threshold (Ct) was set to 200.

### RCA-FL test

The RCA-FL test is an RCA-based fluorescent detection method. It was conducted using the PCR instrument (CFX96 Touch™ Real-Time PCR, Bio-Rad) with a constant temperature (55°C) without thermal cycling. Similar to the ordinary RCA process, probes met target genes, ligation and amplification occurred, and fluorescent signals were detected as time passed. T4 ligase (12.5 M) and Phi29 polymerase (1.25 μM) were used for ligation and amplification, respectively. Since we adopted a specially designed dumbbell-shaped probe, amplified long DNAs tended to easily entangle with one another, aggregating with neighboring DNAs and forming a DNA gel. The synthesized SARS-CoV-2 oligomers (DNA and RNA) were prepared for the probes, which were combined with the target pathogens. For this purpose, ORF1ab, RdRp, N, and E genes were synthesized. For specific binding between the fluorescent dye and amplified products, we used the SYBR Green I dye (Invitrogen) at 1:3000 dilution. Further detailed information can be found elsewhere [14, 15].

### RCA-flow test

For the RCA-flow test, a microfluidic system was adopted, which was developed in our recent study [14]. The system consists of a sample chamber, glass tube, padlock probe-conjugated nylon mesh, and waste chamber with a rubber lid. Because the waste chamber is sealed with a rubber lid, the sample loaded into the sample chamber cannot flow through the test tube. When the rubber lid is connected to the atmosphere, the sample fluid is driven to the waste chamber owing to the elevated hydrostatic pressure in the sample chamber.

In this RCA flow test, we adopted a very thin mesh, on which immobilized padlock probes captured the target genes. Hybridization occurred when the probe encountered the target pathogen. Using a ligase (T4 ligase, 12.5 μM), the opened padlock probe was ligated to form a closed-loop template, which could then undergo the RCA process. As time passed, complementary single-stranded DNA was elongated with a dumbbell shape using Bst 3.0 DNA polymerase during the RCA process. Because of the dumbbell-shaped template, amplified long DNAs tended to easily entangle with one another, aggregating with neighboring DNAs and forming a DNA gel. Consequently, the hydrogel blocked the micropores of the mesh either partially or completely, and thus there was retarded flow or no flow depending on the degree of micropore blocking by the hydrogel. Further detailed information can be found in our previous study [14, 15].

## Results and discussion

### Evaluation of the collection of SAR-CoV-2 from masks

**Fig 2:**
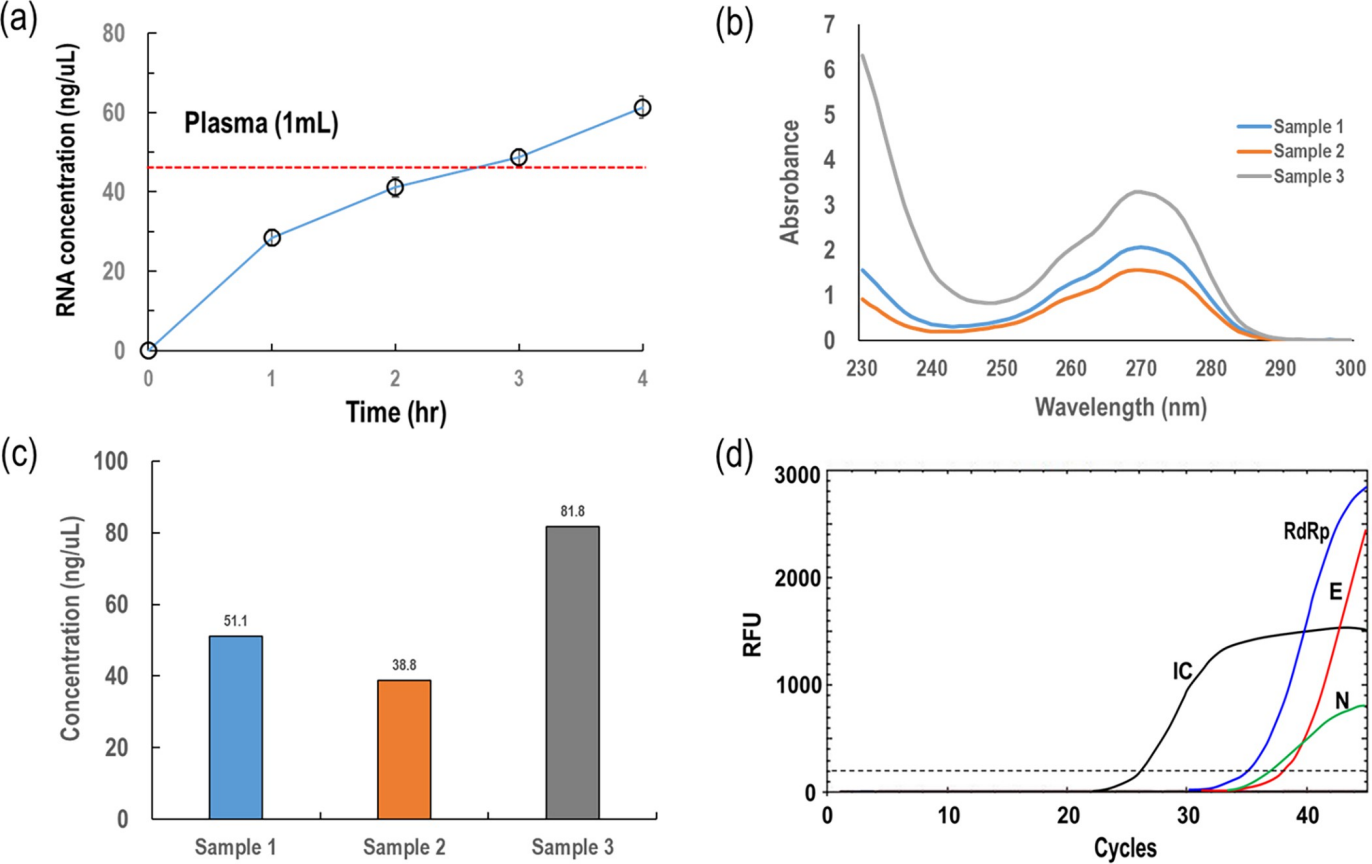
(A) Collected nucleic acid quantity with mask wearing time; (B) absorbance spectrum results for three different masks; (C) concentration of collected RNA from three masks; (D) PCR results of a clinical mask sample.

Prior to examining the masks of patients, we examined the effect of mask wearing time on the quantity of nucleic acids collected from the masks of healthy controls. It is worthy to note that exhaled breath includes various materials such as DNA, RNA, exosomes, and diverse volatile organic compounds [17, 18]. As shown in Fig 2(A), the quantity of extracted RNA increased almost linearly with wearing time, and the amount of DNA extracted from masks worn for more than 3 h was even greater than that from plasma. Thus, we recommended collecting masks that have been worn for more than 3 hours by patients.

Using spectrum analysis, we examined the concentration of the RNA collected from the masks of patients, as shown in Fig 2(B) and 2(C). Depending on clinical samples, there were significant differences among the concentrations, but all of them were in order of tens of nanograms per microliter (ng/μL), which was sufficient for analysis via PCR and NGS. Fig 2(D) shows typical PCR results wherein clinical samples were collected from masks worn by SAR-CoV-2-infected patients. The signal for internal control was first observed at the cycle of 26.3, whereas those for RdRp, N, and E genes were observed at the cycles of 34.4, 36.8, and 37.5, respectively. Thus, the present results with the facial masks were identical to the PCR results with the nasopharyngeal swab sample.

### Evaluation of RCA-FL using mask samples

**Fig 3:**
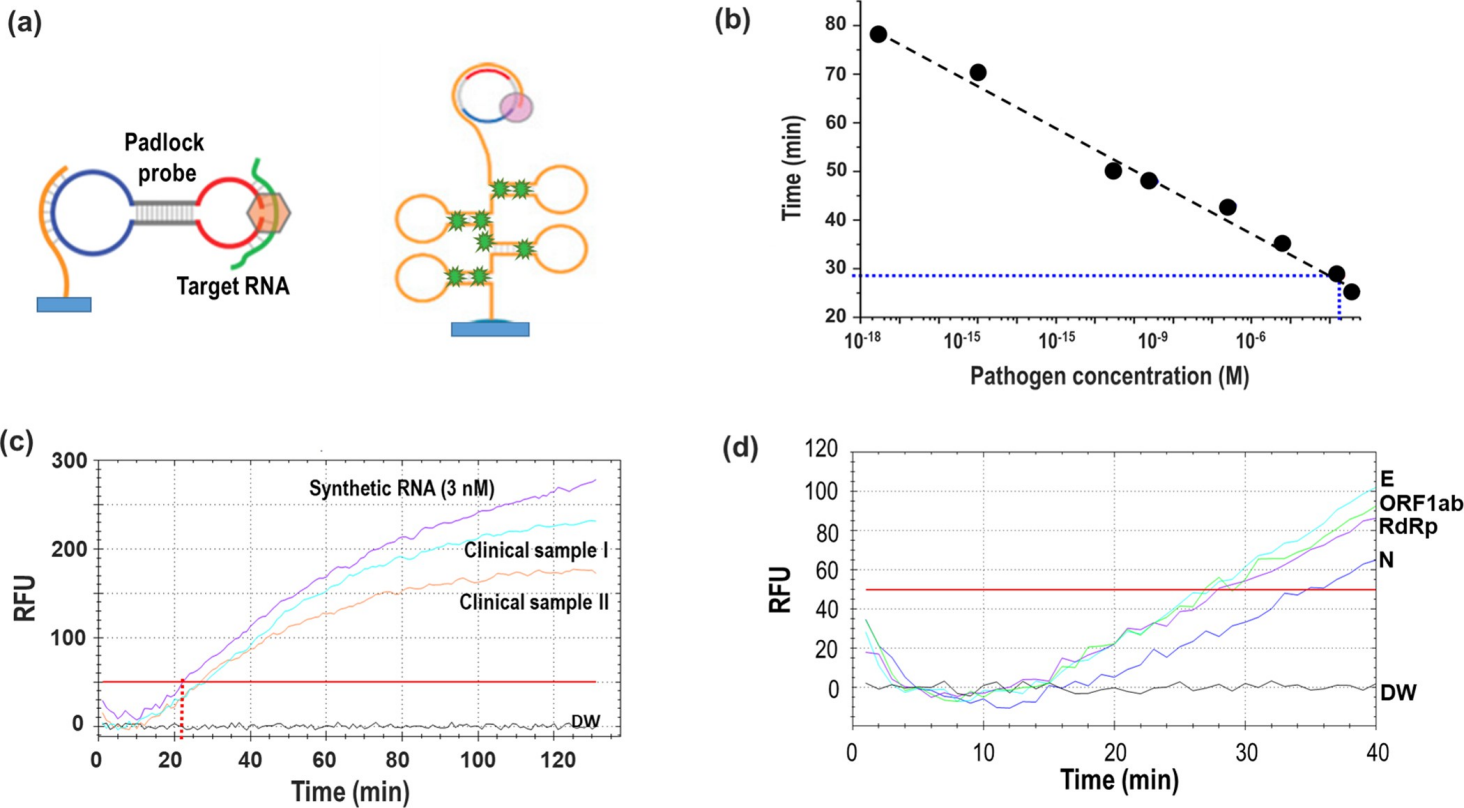
(A) Schematic of RCA using padlock probe; (B) calibration curve for detecting synthesized SAR-CoV-2 RNA with decreasing concentration; (C) comparison of RCA-FL results between synthetic RNA (3 nM) and clinical mask samples; (D) RCA-FLPCR results for four target pathogens from a clinical mask sample.

**Fig 4:**
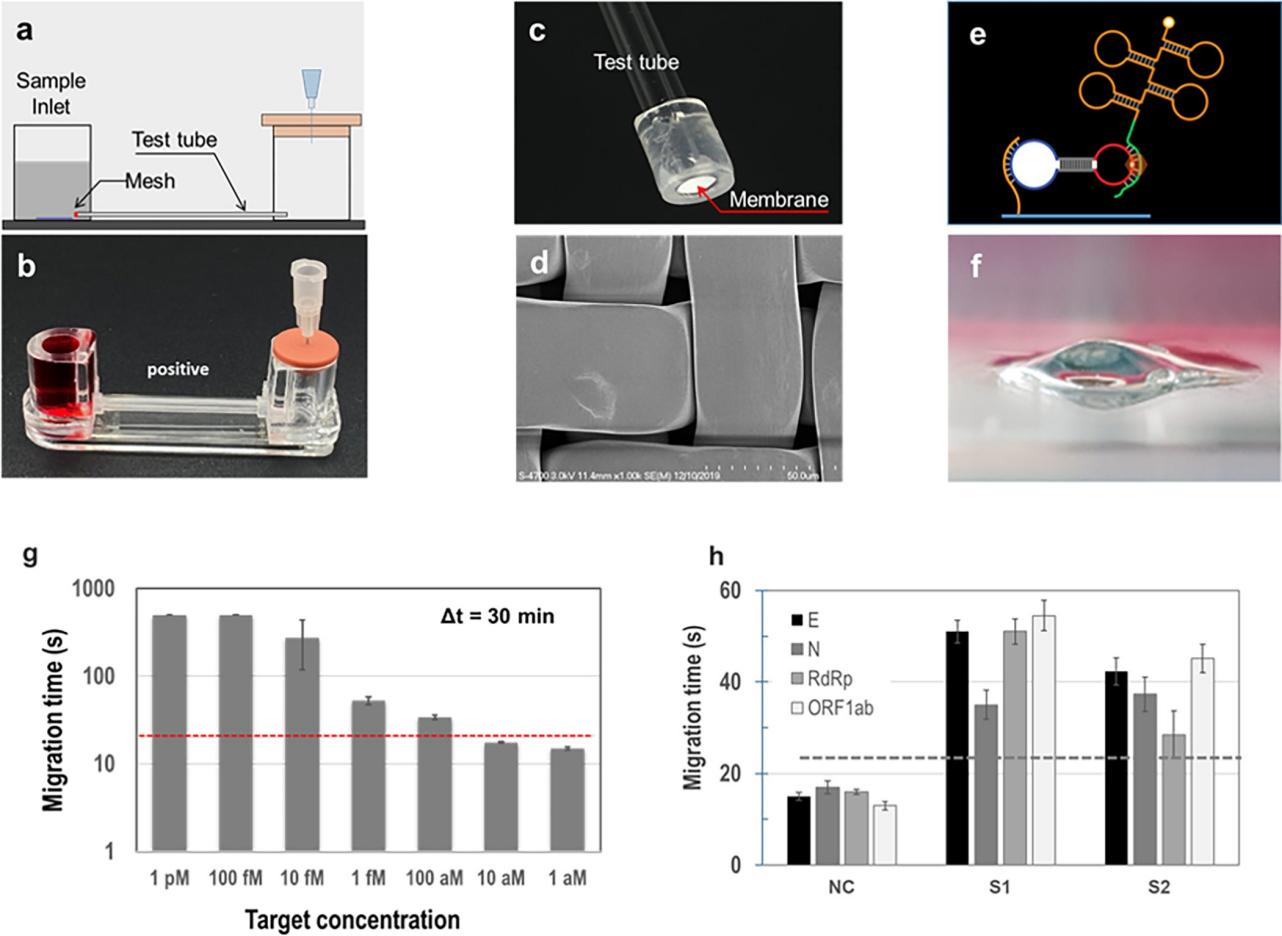
(A) Schematic of RCA-flow with microfluidics; (B) photograph of an integrated microfluidic system with RCA detection for SAR-CoV-2; (C) photograph of a test tube with nylon mesh; (D) SEM photograph of the nylon mesh having microscale pores; (E) schematics of RCA on mesh surface; (F) hydrogel formation through RCA reaction with RNA obtained from clinical mask sample; (G) calibration chart with various concentrations of synthetic RNA; (H) RCA-flow results (migration time) with clinical mask samples.

Fig 3 shows a schematic of the RCA-FL test for detecting SARS-CoV-2 and the corresponding results. As described earlier, a fluorescent signal was detected using a PCR device. For the analysis of pathogen concentrations obtained from mask samples, we performed serial experiments with decreasing concentrations of synthetic RNA (3.13 aM–3 μM). As shown in Fig 3(B), a standard curve was prepared for the synthesized samples at various concentrations. Measurements were conducted up to attomolar concentration levels with a reaction time of approximately 80 min, which were similar to those conducted in our previous reports [5, 9]. Fig 3(C) shows the RCA-FL results for synthetic RNA (3 nM) and those for clinical mask samples with unknown pathogen concentrations and compares them. The synthetic sample was detected at 22.1 min, whereas the two clinical mask samples were detected at 26.9 min and 27.7 min. Thus, the target pathogen concentrations can be roughly estimated to be around hundreds of picomolar concentrations (pM). The threshold times were different for each target gene, as shown in Fig 4(D). In this case, the E-gene was first detected at 25.9 min, whereas the N-gene was last detected at 34.5 min.

### Evaluation of RCA-flow with mask samples

An integrated microfluidic system and its detection results are shown in Fig 4. The microfluidic system can be operated alone without any devices such as syringe pumps or connecting tubes. Because the liquid level of the sample chamber is higher than that of the other chamber, the flow is driven by gravity by puncturing the rubber lid. However, when a target pathogen such as SARS-CoV-2 exists in the sample, hydrogels are generated, which block the micropores in the mesh. Owing to the small size of the micropores (approximately 1 μm) in the nylon mesh, these micropores were rapidly blocked by DNA entanglement through the RCA process. Fig 4(D) shows the SEM images of the nylon mesh at high magnifications, whereas Fig 4(F) shows a photograph of the hydrogel formed through RCA reaction with RNA obtained from the clinical mask sample. It is worth noting that in the absence of pathogens, there is natural flow due to gravity, and the repeated tests showed an excellent coefficient of variation for the migration time (< 5%).

With decreasing pathogen concentration, RCA-flow tests were conducted and the corresponding results, as shown in Fig 4(G), were similar to those in our previous reports [14]. After incubation for 30 min, synthetic RNA was detected down to 100 aM with a threshold time of 21 s, and this LOD result decreased slightly from the previous one (3 aM). It was also found that RNA was slightly less amplified via RCA than DNA. Increased incubation time for RCA would certainly result in a higher LOD and vice versa. Fig 4(H) shows the results of RCA-flow with four different probes for clinical samples. The negative control showed a significantly shorter time since there was no hydrogel formation on the micropores, whereas the clinical samples yielded a prolonged migration time compared with the threshold (21 s). For the S1 sample, E-, RdRp, and ORF1ab genes yielded quite a longer time compared with the N gene, whereas the RdRp gene in the S2 sample yielded a minimum value compared with the others. Thus, the difference in migration time among the four genes was not caused by the sensing capability of a specific gene.

Table 1 summarizes all the results obtained from PCR, RCA-FL, and RCA-flow for the detection of SAR-CoV-2 in mask samples (n = 3). Each test was conducted using three or four different target probes (N, E, RdRp, and ORF1ab). For samples 1 and 2, all three tests showed positive results, showing consistent results. However, for sample 3, PCR and RCA-FL yielded negative results, and one of the RCA-flow tests with the N-gene probe yielded positive results. Assuming that the PCR test was a verified reference test, RCA-flow with an N-gene probe yielded false-positive results. This result might be caused by nonspecific binding between probes and non-target genes and false amplification through the RCA process. This issue is the weakest point of RCA compared with the normal PCR assay, and this is the main issue that needs to be resolved in RCA-based assays [19]. In addition, as shown in Table 1, PCR and RCA-FL yielded negative results for samples 2 and 3, which were obtained from the masks of infected patients worn more than 3 h. These results can be analyzed for one or two reasons. One is a potential error in mask sampling, including mask storage and viral RNA extraction. Second, viral RNA was not captured in a facial mask. The latter has less possibility because other mask samples yielded many RNA with positive assaying results. The former should be carefully considered, and a standard protocol should be established for storage temperature, storage time, and extraction process.

**Table 1:** Fluorescence and microchip results for various genes of SARS-CoV-2 and compared with commercialized kit.

	genes	PCR (Control)	RCA-FL	RCA-Flow
Sample 1	N	**○**	**○**	**○**
	E	**○**	**○**	**○**
	RdRp	**○**	**○**	**○**
	ORF1ab	NA	**○**	**○**
	Readout	**P**	**P**	**P**
Sample 2	N	**○**	**○**	**○**
	E	**○**	**○**	**○**
	RdRp	**○**	**○**	**○**
	ORF1ab	NA	**○**	**○**
	Readout	**P**	**P**	**P**
Sample 3	N	X	X	○*
	E	X	X	X
	RdRp	X	X	X
	ORF1ab	NA	X	X
	**Readout**	**N**	**N**	**N**

○: detected; X: not detected; P: positive; N: negative; NA: not available; *false positive

Through the present study, we proposed the mask as a virus collecting tool, which can make the present complex and inconvenient sample collection protocol easy and simple. In other words, the original objective of masks that is to filter external airborne particulates has been changed to collect respiratory particulates including viruses. Since we adopted a single type, certified KF94 masks, there was no chance examine the effects of mask materials and structure on collection efficiency of respiratory viruses. Typical factors are the porosity and electrostatic strength of the mask, which is very worthy of study. Of course, these virus collection efficiency would be highly dependent on physicochemical properties of respiratory droplets [20, 21].

The present study demonstrates that the respiratory viruses from an infected person can be effectively collected with a certified face mask such as KH94 or N95. The results of the present study imply how important personal protective equipment (PPE) is in blocking the spread of infectious viruses and in personal quarantine. Many studies reported that face mask could block the respiratory aerosols [2, 3, 5] but the present study confirmed that respiratory viruses were collected in face mask. In other words, respiratory viruses that are continuously released to the outside of the infected person through breathing can be effectively trapped by the mask. Thus, under pandemic situation, wearing a mask is not only beneficial for protecting individuals from the environment, but also essential to stop the spread of the virus for neighbors and public health.

## Conclusion

We investigated the potential use of facial masks for collecting viruses from exhaled breath and detecting SARS-CoV-2 infection. In this study, facial masks were proven to collect viruses from the exhaled breath of infected patients, and they were sufficient for virus detection using PCR and RCA tests. Depending on the wearing time, the extracted quantities of nucleic acids from masks could be higher than those from plasma. Thus, the present study confirmed that facial masks can be used for virus sample collection, and these samples are equivalent to nasopharyngeal swab (NPS) samples. Owing to the nature of masks, sampling of exhaled breath using facial masks could be the most noninvasive, simple, and easy method to collect viruses. Furthermore, we confirmed that the RCA-flow adopting a highly integrated microfluidic system detected SAR-CoV-2 as sensitively as the PCR test.

However, in the present study, some problems of mask sampling were identified, which require a solution. It is necessary to develop a new method for effectively extracting viral RNA from masks, which is quite different from other samples such as NPS and plasma. Direct collection and sensing of viruses in facial masks would be highly recommended. More importantly, the RCA test may yield false positives, therefore, more specific target binding should be developed for precision diagnosis. As a limitation of the present study, the mask samples were not compared with other biological fluids, such as saliva, mucus, and sputum, except NPS. In addition, the number of clinical samples was limited as a proof of concept.

## Supporting information

S1 File (DOCX)
