# Supplementary material for: Collection and detection of SARS-CoV-2 in exhaled breath using face mask
Source: PLoS One. 2022 Aug 18;17(8):e0270765. doi: 10.1371/journal.pone.0270765 (PMC9387863; doi:10.1371/journal.pone.0270765)
Supplement: S1 File — (DOCX) [file pone.0270765.s001.docx]

**Supplementary Information**

Collection and detection of SARS-CoV-2 in exhaled breath using face mask

Hwang-soo Kim^1¶^, Hansol Lee^2¶^, Junsoo Park^1^, Naseem Abbas^3^, Seonghui Kang^4^, Hakjun Hyun^5^, Hye Seong^5^, Jin Gu Yoon^5^, Ji Yun Noh^5^, Woo Joo Kim^5^*, Sehyun Shin^1,6^*

*^1^ Department of Micro-nano System Engineering, Korea University, Seoul, 02841, Republic of Korea*
*^2^ Asia Pacific Influenza Institute, Korea University College of Medicine, Seoul, 02841, Republic of Korea*

*^3^ Department of Mechanical Engineering, Sejong University, Seoul,05006, Republic of Korea*

*^4^ Division of Infectious Diseases, Department of Internal Medicine, Konyang University Hospital, Daejeon, 35365, Republic of Korea*

*^5^ Division of Infectious Diseases, Department of Internal Medicine, Korea University College of Medicine, Seoul, 02841, Republic of Korea*
*^6^ School of Mechanical Engineering, Korea University, Seoul, 02841, Republic of Korea*

**List of Supplementary Table Caption**

**Table S1.** List of SARS-CoV-2 template, pathogen, linker primer and primer nucleic acid sequences used in this study

**List of Supplementary Figure Captions**

**Fig. S1** The process of extracting RNA from the collected mask.

**Fig. S2** Verification of nucleic acid extraction amount according to SARS-CoV-2 nucleic acid extraction method.

**Fig. S3** SARS-CoV-2 Detection Using DNA Hydrogel Formation and Fluorescence.

**Fig. S4** Photographs of microfluidic kits for the detection of SARS-CoV-2..

Table S1. List of SARS-CoV-2 template, pathogen, linker primer and primer nucleic acid sequences used in this study

| Strands | Sequence (5’ → 3‘) |
| --- | --- |
| COVID-19  N gene template  (102 nt) | 5’-phosphate-AAT ACC ATC TT A ATC GAA GTA CTC AGC GTA AGT TTA GAG GTA GCA TGC TAG TAT CGA CGT CCC ACG TAC CAA CTT ACG CTG AGT ACT TCG ATT GGT AGT AGA-3’ |
| COVID-19  E gene template  (102 nt) | 5’-phosphate-CAC GTT AAC AAA ATC GAA GTA CTC AGC GTA AGT TTA GAG GTA GCA TGC TAG TAT CGA CGT CCC ACG TAC CAA CTT ACG CTG AGT ACT TCG ATT TAC AAG ACT-3’ |
| COVID-19  RdRp gene template  (102 nt) | 5’-phosphate-GAA CTT CCT TCA ATC GAA GTA CTC AGC GTA AGT TTA GAG GTA GCA TGC TAG TAT CGA CGT CCC ACG TAC CAA CTT ACG CTG AGT ACT TCG ATT AAT TCA ACA-3’ |
| COVID-19  ORF1ab gene template  (102 nt) | 5’-phosphate-GTA GCC ATA CTA ATC GAA GTA CTC AGC GTA AGT TTA GAG GTA GCA TGC TAG TAT CGA CGT CCC ACG TAC CAA CTT ACG CTG AGT ACT TCG ATT AAG TAG TAT-3’ |
| Covid-19 pathogen(26 nt)_ N gene | 5’- TAA AAG ATG GTA TTT CTA CTA CCT TA-3’ |
| Covid-19 pathogen(26 nt)_ E gene | 5’- TAA TTG TTA ACG TGA GTC TTG TAT TA-3’ |
| Covid-19 pathogen(26 nt)_ RdRp gene | 5’- TAA GAA GGA AGT TCT GTT GAA TT TTA-3’ |
| COVID-19 pathogen  (26 nt) | 5’- TAA AGT ATG GCT ACA TAC TAC TT TTA-3’ |
| NH2-polyA-primer  (32 nt) | 5’-Amino(C6)-AAA AAA AAA GGG ACG TCG ATA CTA GCA TGC TA-3’ |
| Additional Primer  (12 nt) | 5’-TGC TAG TAT CGA-3’ |

*Red color indicates the pathogen binding site, green indicates the self-assembly region, and blue indicates the primer binding site.


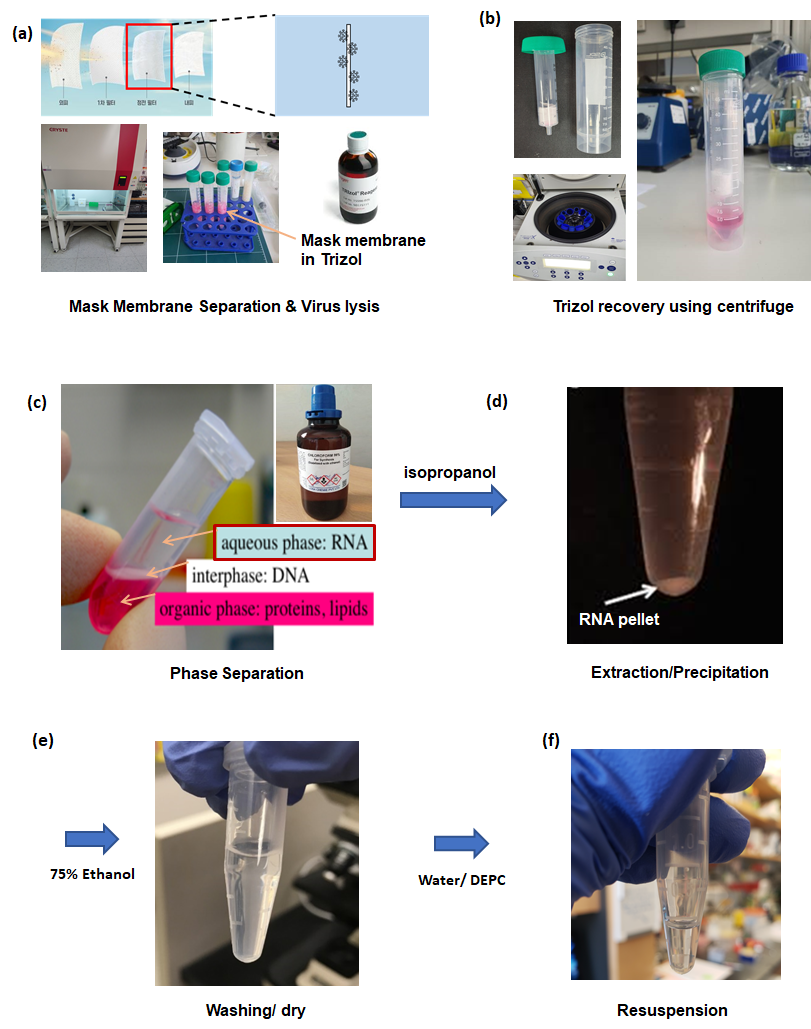


**Fig. S1** The process of extracting RNA from the collected mask. (a) Put the cut mask membrane in Trizol and mix well. (b) Recover all the solutions to the mask membrane using a centrifuge. (c) Chloroform was dispensed into the tube, the mixture extracted from the mask was added, and incubated. (d) After centrifugation using a centrifuge, only the transparent supernatant is separated. (e) After mixing isopropanol with the supernatant, the mixture is stirred and incubated at room temperature. (f) After centrifuging the sample again, the supernatant is removed, and ethanol is added to the RNA, washed and dried. (g) After dispensing and mixing RNase-free water (DW), the mixture was incubated in a heating block to extract RNA.

**Validation of RNA yield according to mask nucleic acid extraction and recovery method**

In order to confirm the optimal extraction method for SARS-CoV-2, the column method and the precipitation method were simultaneously reviewed and the results were compared. The column method significantly shortened the extraction time compared to the process time, but the concentration of extracted nucleic acid was low, whereas the precipitation method had better nucleic acid extraction concentration and purity, but took a lot of processing time. (Fig. S2(a) and S2(b)).


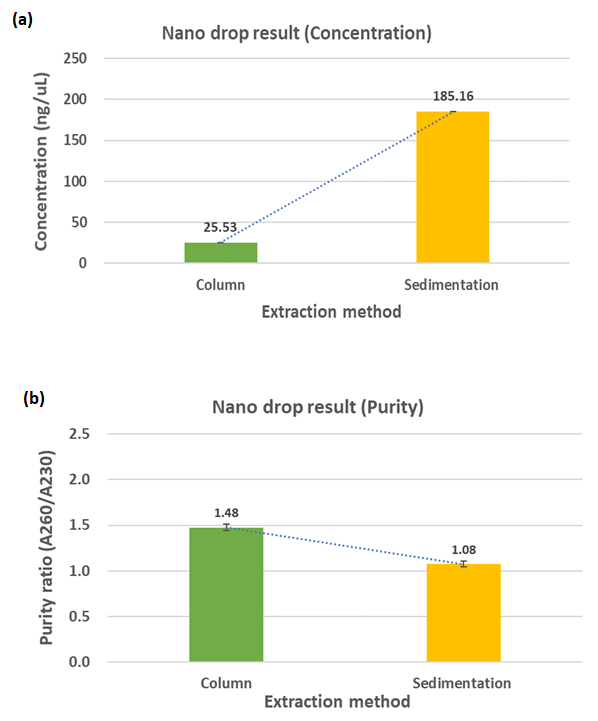


**Fig. S2** Verification of nucleic acid extraction amount according to SARS-CoV-2 nucleic acid extraction method. (a) Comparison of the concentration of extracted SARS-CoV-2 nucleic acid according to the extraction method, (b) Purity comparison of extracted SARS-CoV-2 nucleic acids according to the extraction method.

**Verification of SARS-CoV-2 detection by RCA reaction**

We examined the fluorescence detection method for the DNA hydrogel formation associated with RCA reaction. While RCA reaction was proceeding in a PCR tube, fluorescence signal was monitored using a real-time PCR system (CFX96 Touch™ Real-Time PCR, Bio-Rad). For the florescence signal, we adopted an intercalated dye (SYBR™ Green I green). While allele-specific amplification was performed, fluorescence signal was monitored as shown in Fig. S3.

**
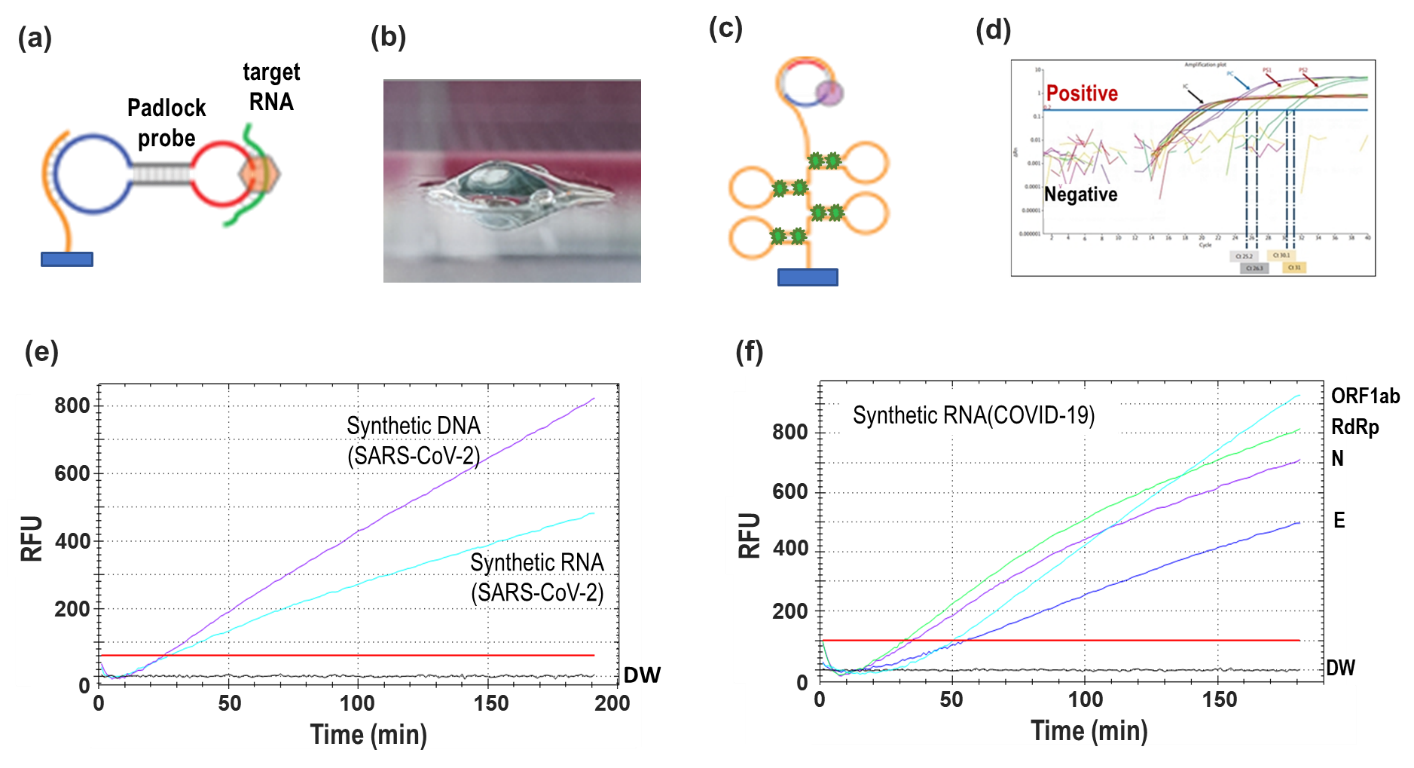
**

**Fig. S3** SARS-CoV-2 Detection Using DNA Hydrogel Formation and Fluorescence. (a) Schematic of hydrogel formation via rolling circle amplification, (b) DNA hydrogels formed via RCA, (c) Schematic diagram of fluorescence detection via rolling circle amplification, (d) Images of Real-Time PCR detection via RCA, (e) Real-Time PCR Fluorescence Detection Using Synthesized Target DNA and RNA of SARS-CoV-2 and (f) Real-Time PCR Fluorescence Detection Using Various Target Genes of Synthesized SARS-CoV-2 Target RNA.

**Comparison of negative control and detection of each SARS-CoV-2 target gene**

To confirm the detection of SARS-CoV-2, the nucleic acids extracted from the infected patient masks were put into a sample reservoir, in which each nylon mesh was conjugated with specific padlock probe such as N gene, E gene, RdRp gene, ORF1ab gene. RCA reaction was performed on the nylon mesh for 30 min and then RCA-flow test was performed, as shown in Fig S4 (a). For negative control sample (no RNA for SARS-CoV-2), there was direct flow to the other with rubber cap puncture (Fig S4 (b)). For positive samples, as shown Fig S4 (c-f), there was no flow even after rubber cap puncture.


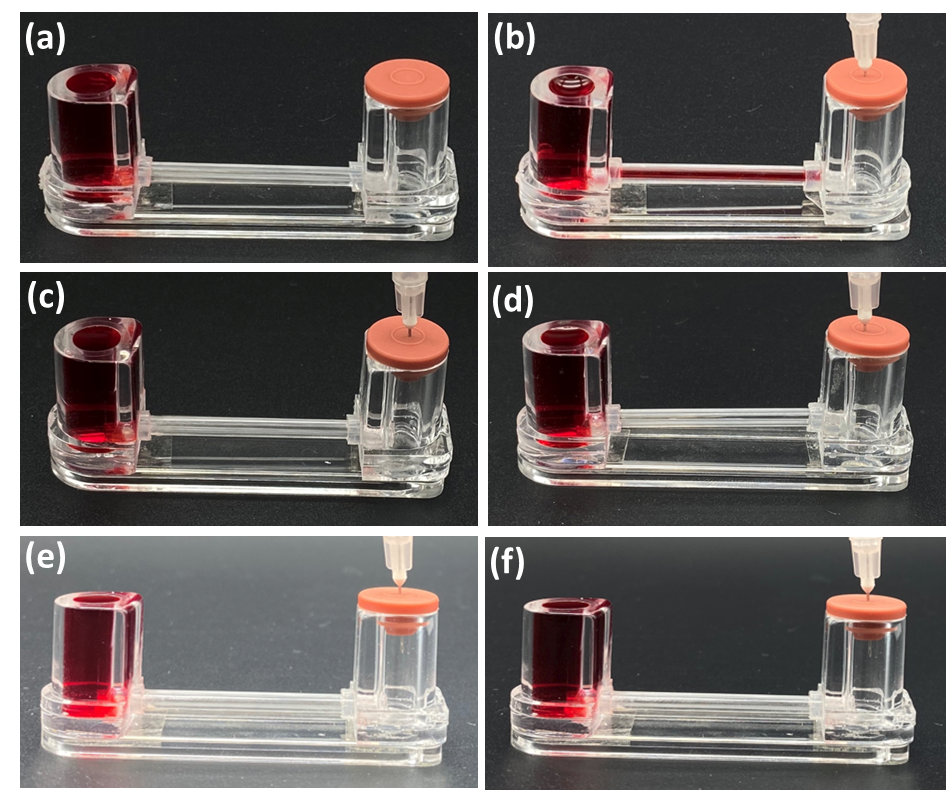


**Fig. S4** Original images of microfluidic kits for the detection of SARS-CoV-2. (a) after incubation (b) Covid-19 no-pathogen (negative control), (c) N gene, (d) E gene, (e) RdRp gene and (f) ORF1ab gene.
